# Monitoring hypertensive disorders in pregnancy to prevent preeclampsia in pregnant women of advanced maternal age: Trial mimicking with retrospective data

**DOI:** 10.1515/med-2022-0560

**Published:** 2022-11-22

**Authors:** Yali Deng, Lifei She, Xiaoye Li, Weisi Lai, Ling Yu, Wen Zhang, Yanting Nie, Songyuan Xiao, Hongyu Liu, Yang Zhou, Ting Luo, Wen Deng, Jinyu Liu, Xihong Zhou, Ying Wen, Yanhong Zhong, Lingyi Xiao, Yiling Ding, Mei Peng

**Affiliations:** Department of Gynaecology and Obstetrics, The Second Xiangya Hospital of Central South University, Changsha 410011, P.R. China; Department of Pharmacy, The Maternal and Child Health Hospital of Hunan Province, Changsha 410000, P.R. China; Department of Gynaecology and Obstetrics, Sanya Central Hospital (Hainan Third People’s Hospital), Sanya 572000, P.R. China; Department of Gynaecology and Obstetrics, Maternal and Child Health Hospital in Yuanjiang City, Yuanjiang 413111, P.R. China; Department of Gynaecology and Obstetrics, Affiliated Hospital of Xiangnan University, Chenzhou 423000, P.R. China; Department of Gynaecology and Obstetrics, The Second Xiangya Hospital of Central South University, No. 139 People’s Middle Road, Changsha 410011, P.R. China

**Keywords:** pregnant women at advanced maternal age, low-molecular-weight heparin, aspirin, preeclampsia

## Abstract

This study investigated the implication of monitoring hypertensive disorders in pregnancy (HDP) to prevent preeclampsia (PE) in pregnant women of advanced maternal age. Between January 2016 and April 2021, 262 consecutive pregnant women aged ≥40 years were recruited. Extensive monitoring of hypertensive disorders in pregnancy, including blood hypercoagulability screening and subsequent interventions, was performed in 129 pregnant women in our university hospital. The remaining 133 patients from other centres, who did not receive antenatal maternal pregnancy screening and preventive intervention during the same period, constituted the non-intervention group enabling comparison to mimic a trial. The incidences of hypertensive disorders, mild and severe PE, eclampsia, and chronic hypertension complicated by PE in the intervention group were significantly lower than in the non-intervention group (10.08 versus 20.30%, 8.52 versus 18.80%, 7.75 versus 21.05%, 0 versus 3.01%, and 3.86 versus 15.04%, respectively; *P* < 0.05). Premature birth, low birth weight, and foetal loss were significantly rarer in the intervention group than in the non-intervention group (6.98 versus 24.81%, 7.75 versus 21.80%, and 0.78 versus 14.29% respectively; *P* < 0.001). The comparison of MP with routine blood coagulation biochemical examination found that the MP detection system of Beijing Yes Medical Devices Co., Ltd., had similar sensitivity as thromboelastogram. Still, it was significantly better than the routine biochemical indicators (*P* < 0.01). Based on MP parameters, early anticoagulant treatment with low-molecular-weight heparin or low-dose aspirin in pregnant women with hypercoagulability can effectively prevent the occurrence of PE and significantly improve the prognosis of both mothers and infants.

## 1 Introduction

Gestational de novo hypertension, chronic hypertension, preeclampsia–eclampsia, and chronic hypertension with superimposed preeclampsia (PE) have an incidence of 5–12% among pregnant women. Untreated hypertensive disorders in pregnancy (HDP) increase maternal and perinatal mortality [1].

PE is the de novo onset of elevated blood pressure and proteinuria after 20 weeks of gestation, maternal multiple-organ damage (kidney failure, hepatic, neurological, and haematological complications), and foetal growth restriction (FGR) [1,2]. Active screening of women at high PE risk and early preventive measures must be validated to define an accurate indication.

Since blood hypercoagulability is the pathological basis with the highest evidence level of the onset of PE [3,4,5], it is urgent to monitor hypercoagulability indicators starting in the first trimester. Lidan et al. [6] found that thrombelastography (TEG) provides more accurate information for monitoring the coagulation of patients with PE and can be used as a reliable marker for assessing the severity of PE. Okoye et al. [7] found that reduced levels of plasma protein C and protein S are correlated with the occurrence and development of PE. Other scholars found that abnormal haemorheology and coagulation parameters such as prothrombin time (PT), activated partial thromboplastin time (APTT), or D-dimer are closely correlated with the occurrence of PE [8,9]. However, previous studies were mostly limited to detecting a single parameter with low specificity and sensitivity. Therefore, establishing compound predictive blood hypercoagulability parameters in early pregnancy will have important clinical value for preventing and treating PE early and improving adverse pregnancy outcomes. Finding a simple, fast, sensitive, and clinically effective antenatal method before 20 weeks of gestation or even earlier has been a puzzle for obstetric clinicians.

Based on the above clinical phenomena and confusion, we found that MP in monitoring the hypercoagulable state showed a higher sensitivity than other biochemical indicators. From the perspective of the epidemiology of the disease and based on the fact that the age of ≥40 years is a high-risk factor, we evaluated and managed pregnant women aged ≥40 years providing early monitoring and intervention to explore a simple and easy way to screen for high blood coagulation by measuring a set of parameters with high sensitivity that can provide clinical guidance to minimise the likelihood of developing PE in pregnant women with high-risk factors, to achieve a foetal prognosis that is close to or even at the normal level, and then to provide a convincing clinical basis for the early prediction and early pharmaceutical intervention of the disease.

## 2 Materials and methods

### 2.1 General data

A retrospective analysis of 129 pregnant women aged ≥40 years who sought medical advice in the Obstetric Outpatient Clinic of the Second Xiangya Hospital of Central South University, Changsha, Hunan, starting in early pregnancy from January 2016 to April 2021, was set as the intervention group (with consecutive enrollment). At the first check-up during early pregnancy, coagulation parameters obtained from MP were determined, and blood hypercoagulability indicators, including coagulation function, TEG, haemorheology, plasma protein S, and plasma protein C, were measured after routine blood sampling. Any item suggesting a hypercoagulable state was included in the positive case management group for early intervention, while the normal ones were not regarded as the observation subjects. The latter were rechecked after 1–2 weeks, and the abnormal ones were given specific management until delivery.

In addition, 133 pregnant women aged ≥40 years who were not actively screened for blood hypercoagulability and did not receive intervention within the same period were consecutively included in the non-intervention group. These 133 patients came from other municipal hospitals. The occurrence of HDP and foetal outcomes in the two groups were observed and compared.

This study was reviewed and approved by the Ethics Committee of The Second Xiangya Hospital, Central South University (approval no. (2020) Lun Shen Di (Yan 573)), and all procedures were performed according to the relevant guidelines and regulations. Written informed consent was obtained from each participant.

### 2.2 Inclusion and exclusion criteria

The inclusion criteria were as follows: patients ≥40 years of age; patients whose first visit time was ≤12 weeks after their last menstruation; patients who were aware of this study and signed the consent form; patients whose gestational sac and uterus sizes were in line with the gestational age according to the colour ultrasound examination; and patients with a single foetus.

The exclusion criteria were as follows: patients with a history of drug allergy; patients with severe dysfunction of the heart, liver, kidney, or other organs; patients with poor treatment compliance; and patients with contraindications to aspirin or low-molecular-weight heparin (LMWH).

### 2.3 Methods

#### 2.3.1 Non-intervention group

This group consisted of pregnant women who underwent routine pregnancy examinations but without blood hypercoagulability screening.

#### 2.3.2 Intervention group

Apart from regular pregnancy checks, this group also received the following examinations:

(1) Using the Monitoring Pregnancy (MP) detection system of Beijing Yes Medical Devices Co., Ltd.: noninvasive monitoring of the changes in the elasticity and blood viscosity of the peripheral resistance vessels of pregnant women indirectly reflecting the state of blood coagulability.
(2) MP detection: the subject was instructed to lie on her left side at an inclination of 45°. The MP monitoring probe was placed at the strongest radial artery pulsation. When the pulse wave appeared on the screen and the waveform became stable, the monitor performed calculations and finally reported the haemodynamic indicators, including systolic blood pressure (SBP), diastolic blood pressure (DBP), mean arterial pressure, heart rate, cardiac index (CI), total peripheral resistance (TPR), viscosity (*V*), mean retention time (TM), and waveform factor (*K*). When SBP was ≥130 mmHg, DBP was ≥80 mmHg, *V* was ≥4.5, vascular compliance was ≤1.2, the half-renewal time was ≥23 s, TM was ≥33 s, *K* was ≥0.4, and TPR was ≥1.2 PRU, the result was regarded as a positive screening finding, and the patient who met any of the standards was enrolled in hypercoagulation management. The maximum MP score was 100%.
(3) Blood biochemical parameters to monitor the blood coagulable status: abnormality in any coagulation parameters, including PT, APTT, antithrombin III, D-dimer, TEG, plasma protein S, and plasma protein C, was considered a positive screening finding, and the patient was enrolled in hypercoagulation management.

When MP was positive while chemical analyses did not reveal a problem, the MP finding was treated as the diagnostic outcome.

#### 2.3.3 Treatment methods

Repeated MP was arranged at 1 or 2 weekly intervals according to the degree of blood hypercoagulability. Those who were positive for MP were given anticoagulation therapy in the form of either aspirin or LMWH. The two drugs were given in combination to patients with obvious hypercoagulability. The specific usage was as follows:

For those with gestational age ≤12 weeks, if the total MP score was ≤60%, LMWH 5000 u subcutaneous injection QD was given; if the total MP score was 60–80%, LMWH 5000 u subcutaneous injection QD was alternated with LMWH 5000 µ subcutaneous injection BID; if the total MP score was ≥80%, LMWH 5000 u subcutaneous injection BID was given; and patients with obvious hypercoagulability were rechecked to come back in a week, at which time the dosage was adjusted as appropriate.

For patients with gestational age >12 weeks, if the total MP score was ≤60%, aspirin 50 mg (PO, taken before bedtime) was given; if the total MP score was 60–70%, aspirin 75 mg (taken before bedtime) was given; if the total MP score was 70–80%, aspirin 100 mg (taken before bedtime) was given; and if the total MP score was ≥80%, then aspirin 100 mg (taken before bedtime) + LMWH 5000 iu QD [10] was given. If there was obvious hypercoagulability, the patients were rechecked after 1 week, and the dosage was adjusted as appropriate. Up to 34–36 weeks of gestation, aspirin was stopped immediately if there were signs of labour or symptoms of bleeding to prevent postpartum haemorrhage since the effect of aspirin on platelet haemostasis keeps going for 7–10 days; for patients whose condition still required anticoagulation treatment, LMWH was given.

Aspirin was stopped at ≥36 weeks of gestation, after which LMWH was used with the daily dosage described above. The medication was discontinued 24 h before the termination of pregnancy.

#### 2.3.4 Observation indicators

(1) The pregnancy outcomes between the two groups were compared.
(2) Coagulability parameters of the pregnant women in the intervention group obtained from MP and blood hypercoagulability indicators obtained from routine blood sampling (coagulation function, TEG, haemorheology, plasma protein S, plasma protein C sensitivity) were compared on their sensitivity and (dis)agreement for identifying blood hypercoagulation in the intervention group.

### 2.4 Statistical analysis

Data were analysed with SPSS 22.0 and GraphPad Prism 8.0. Measurement data are presented as the mean ± standard deviation (*x* ± *s*), and the *t*-test and the Mann‒Whitney *U*-test were used to compare the two groups. Count data were compared with the Kruskal‒Wallis *H* and *χ* ^2^ tests. The mean (dis)agreement between the MP and the biochemical hypercoagulation was calculated according to the Bland–Altman method. Values of *P* < 0.05 indicated that the difference was statistically significant.

## 3 Results

### 3.1 Baseline data

There were no statistically significant differences between the two groups in age, the order of the current pregnancy, BMI, gestational week, and systolic blood pressure or diastolic blood pressure during early pregnancy (*P* > 0.05) (Table 1).

**Table 1:** Comparison of general data between the two groups

Group	*N*	Age	Order of the current pregnancy	Body weight	Systolic blood pressure in early pregnancy	Diastolic blood pressure in early pregnancy
Intervention group	129	44.12 ± 2.99	2.91 ± 1.22	22.13 ± 3.02	120.55 ± 15.12	73.32 ± 8.69
No-intervention group	133	43.71 ± 2.96	3.01 ± 1.33	22.39 ± 3.13	121.38 ± 14.84	73.50 ± 9.46
*U* value		7874.00	8027.50	8190.50	8039.50	8270.00
*P* value		0.248	0.355	0.524	0.379	0.614

### 3.2 Comparison of HDP and foetal outcomes

In the intervention group, 26 pregnant women had chronic hypertension (20.16%), including 5 patients (3.88%) with PE, 13 patients had gestational hypertension (10.08%), 11 patients had mild PE (8.53%), and 10 patients had severe PE (7.75%). Eclampsia was not observed in this group.

In the non-intervention group, 28 pregnant women had chronic hypertension (21.05%), including 20 cases (15.04%) of chronic hypertension complicated with mild PE, 27 (20.30%) had gestational hypertension, 25 (18.80%) had mild PE, 28 cases (21.05%) had severe PE, and 4 (3.01%) had eclampsia.

The incidence of chronic hypertension with superimposed PE, gestational hypertension, mild and severe PE, and eclampsia in the intervention group was significantly lower than in the non-intervention group (*P* < 0.05).

In the intervention group, there were 102 full-term births (79.07%) and 9 premature births (6.98%). There were 10 low-birth-weight infants (7.75%), including 2 full-term infants (1.55%) (1 was caused by complete mediastinal malformation and a small placental area and the other was due to an elongated umbilical cord) and 8 premature infants (6.20%). There were 9 cases of induced labour due to foetal malformation (6.98%) and 9 cases of induced abortion due to missed miscarriage in early pregnancy (6.98%). In addition, 1 foetal loss (0.78%) occurred in the middle or late stage of pregnancy due to HDP. In the non-intervention group, there were 65 full-term births (48.87%) and 33 premature births (24.81%). There were 29 low-birth-weight infants (21.80%), including 11 full-term infants (8.27%) and 18 premature infants (13.53%). Nine women underwent induced labour (6.77%) due to foetal malformations, and 86 with missed miscarriages in early pregnancy underwent induced abortion (6.02%). In addition to the cases of induction of labour due to foetal malformations, 19 cases of foetal loss (14.29%) were due to HDP in the middle or late stages of pregnancy, of whom 7 infants died because of light birth weight and cessation of rescue efforts; 10 infants were induced due to small size for gestational age, the lack of umbilical blood flow, and severe FGR; and 2 babies were delivered by caesarean section because of severe PE complicated by placental abruption and intrauterine foetal death (Table 2).

**Table 2:** Comparison of the onset of preeclampsia and foetal outcome between the intervention group and the non-intervention group

Item	Intervention	Non-intervention	*χ* ^2^	*P* value	OR	95% CI
*n*	129	133				
Chronic hypertension (*n*)	26	28	0.032	0.858	0.947	(0.526–1.747)
Gestational hypertension (*n*)	13	27	5.291	0.021	0.440	(0.221–0.868)
Mild preeclampsia	11	25	5.827	0.016	0.403	(0.183–0.853)
Severe preeclampsia	10	28	9.342	0.002	0.315	(0.154–0.670)
Eclampsia	0	4	3.940	0.048	0.000	(0.000–1.031)
Chronic hypertension with superimposed preeclampsia	5	20	9.452	0.002	0.228	(0.091–0.601)
Full-term birth	102	65	25.841	<0.001	3.952	(2.282–6.708)
Premature birth	9	33	15.481	<0.001	0.227	(0.103–0.486)
Low-birth-weight infant	10	29	10.211	0.001	0.301	(0.148–0.636)
Foetal loss in the middle or late stage of pregnancy	1	19	16.951	<0.001	0.047	(0.004–0.271)
Induced labour due to foetal malformation	9	10	0.029	0.866	0.923	(0.383–2.403)
Induced abortion due to missed miscarriage	9	8	0.100	0.752	1.172	(0.469–3.113)

Data were analysed with GraphPad Prism 8.0. A four-cell *chi-square* table was used to perform the frequency test. OR, odds ratio; CI, confidence interval.

The outcome of the foetuses was significantly better in the intervention group than in the non-intervention group.

### 3.3 Comparison of sensitivity between MP and routine blood hypercoagulation biochemical indicators in monitoring blood hypercoagulability

By comparing the positive rates of MP and blood hypercoagulation biochemical indicators in the intervention group, the sensitivity of MP for monitoring the hypercoagulable state of blood was slightly higher than that of TEG, but this was not statistically significant (OR = 1.284, 95% CI [0.716–2.242]; *P* > 0.05). However, the sensitivity of MP for monitoring HDP was significantly better than that of blood biochemical hypercoagulability indicators (coagulation function, haemorheology, plasma protein C, plasma protein S; *P* < 0.001; see Table 3).

**Table 3:** Comparison of sensitivity between MP and routine blood hypercoagulation biochemical indicators in monitoring blood hypercoagulability in the prevention group

Detecting method	+	–	Positive value (%)	*χ* ^2^ value	*P* value	OR	95% CI
MP monitoring system	100	29	77.52				
TEG	94	35	72.87	0.748	0.387	1.284	(0.716–2.242)
Coagulation function	53	76	41.09	35.481	<0.001	4.945	(2.861–8.341)
Haemorheology	47	82	36.43	44.415	<0.001	6.016	(3.452–10.26)
Plasma protein C	42	87	32.56	52.691	<0.001	7.143	(4.054–12.36)
Plasma protein S	41	88	31.78	54.441	<0.001	7.401	(4.190–12.86)

Data were analysed with GraphPad Prism 8.0. OR, odds ratio; CI, confidence interval.

## 4 Discussion

The incidence of HDP in pregnant women is approximately 5–12%, and HDP is one of the major causes of morbidity and mortality in pregnant women and perinatal infants [11]. Although there have been many studies, the aetiology is still not completely clear. Nonetheless, it is generally agreed that there is primarily a pathological hypercoagulable state and thrombosis tendency. Endothelial injury and a hypercoagulable blood state pathologically lead to vascular complications. Systemic arteriole spasm is considered the primary pathological change in PE. The progression of the disease can lead to increased peripheral resistance and reduced organ blood perfusion, tissue hypoxia, and ischaemia, causing damage to the mother and baby [12,13].

In recent years, the role of epigenetic changes in the development of PE has been suggested, such as altered expression of selective miRNAs, which may play a vital role in both placenta-induced diseases, such as PE and FGR [14,15]. With the arrival of the era of molecular medicine and the need to find a satisfactory set of biomarkers, many researchers and scholars are attempting to create a unique and powerful biochemical model for predicting PE through serum markers. However, predicting early biochemical patterns in serum falls short of the desired biomarker [16,17]. Unfortunately, the biomarkers in maternal peripheral blood used for early screening or diagnosis are unfeasible for large samples in areas with an underdeveloped social economy. Instead, a relatively simple and feasible screening model with rapid results and low detection costs is needed. MP potentially fulfils this clinical demand. For areas or populations with limited budget options, MP may play an efficient role in early prediction and screening because MP is the final state of multifactorial traits (a total of 19 indicators), and its early prediction is even more powerful than pooled biomarkers [18,19,20].

Based on the principle of elastic tubes in haemodynamics, according to which the amount of cardiac output mainly depends on the arterial waveform and pulse pressure difference, and the shape of the pulse wave reflects the specific changes in elasticity of human peripheral resistance blood vessels and blood viscosity, Cong et al. [18] used the MP monitoring system to monitor the pulse wave of the radial artery. After the waveform became stable, changes in a series of parameters, such as CI, peripheral resistance, waveform coefficient, and waveform shape, were observed to clarify the patient’s vascular status in terms of blood pressure, blood viscosity, vascular resistance, vascular wall elasticity, etc. The method proved simple, easy to implement, non-invasive, and fast. Although some scholars have monitored pregnant women using the MP system and achieved good predictions, this monitoring and prediction were carried out after 20 weeks of gestation [19,20]. In the present study, we adopted the MP monitor to detect the arterial pulse wave in early pregnancy, non-invasively monitored the haemodynamic changes in pregnant women of advanced maternal age, and intervened promptly by administering anticoagulation and antithrombotic treatment. That is, a timely and reasonable intervention was given in each period of hypercoagulability rather than waiting until the blood pressure had begun to rise after 20 weeks of pregnancy. We believe that early MP and hypercoagulability monitoring, as well as early intervention once abnormality is observed, can significantly reduce the incidence of PE and its complications in pregnant women of advanced age, improving the prognosis of both mother and baby, and yielding more satisfactory results. The MP monitoring system is a kind of “early warning” platform for the early prediction of blood coagulation in pregnant women of advanced maternal age. The MP monitoring system is simple and easy to operate, has a high positivity rate and good repeatability, and can provide hypercoagulable parameters in early pregnancy to prevent and effectively intervene against PE.

Some scholars have concluded, from comparisons of the values of uterine artery S/D, PI, and RI at 20–24 weeks of gestation, that 22–24 weeks is the best time to predict PE, and after that, appropriate interventions should be taken to prevent it [21]. However, in clinical practice, the pathophysiological process of PE typically ends by pregnancy week 20. At that time, typical clinical manifestations appeared in some patients. Therefore, the time when the uterine artery blood flow and umbilical blood flow are abnormal is often the occurrence and developmental stage of PE, not the initial stage, and the predicted gestational week is close to the PE onset time, which is often after the best time window for preventive and early interventions. Previous studies reported that vascular reconstruction generally starts at gestation week 8 and is virtually completed before 20 pregnancy weeks [22,23,24]. This means that the best period for early intervention and correction of placental and maternal vascular dysfunction has been missed. At the same time, there was an excellent opportunity to improve the prognosis of the foetus. Accordingly, using ultrasound to monitor the blood flow parameters of the uterus and its blood vessels, aiming to predict the occurrence of PE early in the second trimester, may neglect the early stage of the disease. Therefore, clinically, early prediction and early screening should be carried out in the infiltration stage of the myometrial spiral arterioles by trophoblast cells, and intervention should be given before the completion of the vascular reconstruction. This may have a more significant clinical impact and apply to more patients than late screening and intervention.

In the present study, early pregnancy blood hypercoagulability screening and intervention for pregnant women with advanced maternal age yielded significantly better maternal and infant outcomes than controls without such intervention. This finding is in line with a study published in the *New England Journal of Medicine* by Rolnik et al. [25]. Their multicentre randomised controlled study involved 1,620 patients, and they found that starting aspirin 150 mg daily in high-risk pregnant women between 11 and 14 gestation weeks reduced the risk of PE by 82%. Aspirin is an antiplatelet drug with antipyretic, analgesic, and anti-inflammatory effects. The mechanism of its action is mainly to reduce endothelial cell damage, inhibit platelet aggregation and thrombosis, lower blood pressure, and block coagulation [26,27]. Low-dose aspirin does not increase the incidence of maternal thrombocytopenia, maternal postpartum haemorrhage, or the incidence of prenatal or postpartum haemorrhage, nor does it increase neonatal bleeding tendency and neonatal morbidity. A low aspirin dosage is safe for the mother and baby [28,29,30].

LMWH is heparin fragments obtained by chemical decomposition or enzymatic cleavage of unfractionated heparin. The molecular weight of LMWH ranges from 4,000 to 6,500 µ. LMWH is an antithrombin-dependent thrombin inhibitor. It shows a stable dose–effect relationship in the body, and it is large enough that it does not pass to the placenta and has no teratogenic effect on the foetus. In addition to the anticoagulation and inhibition of platelet aggregation, LMWH can play anti-inflammatory, antiapoptotic, and antithrombotic roles and can protect vascular endothelial cells; it can reduce blood viscosity, improve placental function, increase foetal blood supply, and increase foetal intrauterine reserve capacity, thereby promoting the foetal growth [31]. As early as the 1960s, scholars found that LMWH had a positive therapeutic effect on the complications of PE [32]. LMWH plays a critical role in the invasion and proliferation of early trophoblast cells [33], enhancing the pathological mechanism of “shallow placental implantation” in PE. It does not affect the coagulation function of the foetus, does not increase the incidence of neonatal pathological jaundice, is safe to use during pregnancy, and has few adverse reactions. The most common adverse reaction to LMWH is an increased bleeding tendency. However, the incidence of bleeding complications from subcutaneous injection of regular doses of LMWH during pregnancy is very low; the incidence of heparin-induced thrombocytopenia is below 1%, and clinically significant osteoporosis is also extremely rare [34,35,36].

The findings of Paolino et al. showed that foetal loss was related to the decline in Treg cells [37]. Early studies have also found that a shortage of Treg cells can lead to embryo implantation failure [38], and the number of Treg cells in peripheral blood and decidua declines during PE [39,40]. Studies have found that both LMWH and aspirin may affect the function of T lymphocytes [41,42], so the two drugs can regulate the function of T lymphocytes while removing the hypercoagulable state of the blood, thereby significantly reducing the occurrence of PE and promoting a good pregnancy outcome. Its specific mechanism of action needs further study.

The present study has some limitations. A small number of cases were analysed in this study, which might account for the difference in induced abortions due to missed foetal abnormalities between the intervention group and the non-intervention group. The two groups of pregnant women were not enrolled simultaneously, so the results may also have certain deviations. We mimicked a trial by comparing patients referred to a tertiary university hospital with those referred to municipal hospitals without randomisation. This may have introduced a selection bias towards our intervention group’s high-risk and more complex cases. The latter may have resulted in underestimating the benefit of early screening and preventive measures. A large-sample, multicentre, double-blind, randomised controlled trial is needed to confirm further – at a higher level of evidence – the superiority of the application of the MP system for early screening of the hypercoagulable state of pregnant women of advanced maternal age and for guiding medication to prevent the occurrence of PE and its complications.

To conclude, the MP monitoring system can be used to detect a hypercoagulable state in advanced-age pregnant women before the presence of clinical signs and symptoms of HDP. MP is non-invasive, easy to use, and low cost. Stratified management and stratified anticoagulant therapy based on MP monitoring results can effectively prevent the occurrence of HDP in older pregnant women predisposed to hypercoagulability and foetal FGR, and premature birth and loss. It noticeably improved maternal–neonatal prognoses. In addition, MP has higher sensitivity for monitoring hypercoagulability than traditional clinical and biochemical indicators. This approach is expected to yield better results, especially for pregnant women of poor socioeconomic status and those living in remote areas, where considerable benefits are expected of early MP application.
